# Improvement of Crystal Identification Accuracy for Depth-of-Interaction Detector System with Peak-to-Charge Discrimination Method

**DOI:** 10.3390/s23104584

**Published:** 2023-05-09

**Authors:** Kento Miyata, Ryo Ogawara, Masayori Ishikawa

**Affiliations:** 1 Graduate School of Biomedical Science and Engineering, Hokkaido University, N-15 W-7 Kita-ku, Sapporo 060-8638, Japan; 2 Institute for Chemical Research, Kyoto University, Gokasho, Uji 611-0011, Japan; ogawara.ryo.7s@kyoto-u.ac.jp; 3 Faculty of Health Sciences, Hokkaido University, N-12 W-5 Kita-ku, Sapporo 060-0812, Japan

**Keywords:** depth of interaction (DOI), position-sensitive photomultiplier tube (PS-PMT), pulse shape discrimination (PSD), small animal positron emission tomography (PET), field programmable gate array (FPGA), data acquisition (DAQ)

## Abstract

In positron emission tomography (PET), parallax errors degrade spatial resolution. The depth of interaction (DOI) information provides the position in the depth of the scintillator interacting with the γ-rays, thus reducing parallax errors. A previous study developed a Peak-to-Charge discrimination (PQD), which can separate spontaneous alpha decay in LaBr_3_:Ce. Since decay constant of GSO:Ce depends on Ce concentration, the PQD is expected to discriminate GSO:Ce scintillators with different Ce concentration. In this study, the PQD-based DOI detector system was developed, which can be processed online and implemented in PET. A detector was composed of four layers of GSO:Ce crystals and a PS-PMT. The four crystals were obtained from both the top and bottom of ingots with a nominal Ce concentration of 0.5 mol% and 1.5 mol%. The PQD was implemented on the Xilinx Zynq-7000 SoC board with 8ch Flash ADC to gain real-time processing, flexibility, and expandability. The results showed that the mean Figure of Merits in 1D between four scintillators are 1.5, 0.99, 0.91 for layers between 1st–2nd, 2nd–3rd, and 3rd–4th respectively, and the mean Error Rate in 1D between four scintillators are 3.50%, 2.96%, 13.3%, and 1.88% for layers 1, 2, 3, and 4, respectively. In addition, the introduction of the 2D PQDs resulted in the mean Figure of Merits in 2D greater than 0.9 and the mean Error Rate in 2D less than 3% in all layers.

## 1. Introduction

Small animal positron emission tomography (PET) is used to visualize and study various biological processes in small laboratory animals such as mice and rats [1]. Common examples of the biological process include neuroreceptor imaging [2], metabolic measurement [3], cancer studies [4], gene expression [5], and drug development [6]. To depict the detailed anatomy of small animals, fine spatial resolution is required.

To improve the spatial resolution of PET, administering nuclides with a shorter range of positrons, using a fine pitch detector, and decreasing non-collinearity of annihilation gamma rays can be taken [1,7]. The two annihilation gamma rays have an angular deviation of 180 ± 0.25° in water [7]. It causes the Gaussian blur in the reconstructed images and degrades spatial resolution. The larger the PET detector ring diameter, the larger the FWHM of the Gaussian blur [1,7]. That is the reason why a smaller ring diameter is preferred, however, it causes parallax errors and degrades the reconstructed image quality, especially at the edge of the ring.

The depth of interaction (DOI) PET reduces parallax errors by using the depth information where the annihilation gamma rays interact with the crystal. Time-of-flight (TOF) information also reduces the positioning uncertainty, increases the signal-to-noise ratio, and thus improves the image quality, however, DOI information is still needed due to limited timing resolution, especially in long narrow crystals. In addition, DOI information can improve the timing resolution of TOF PET [1].

To obtain DOI information, there are a lot of methods such as the pulse shape discrimination (PSD) [8,9,10,11], light sharing [12,13,14,15,16], dual-ended readout [17,18,19], sub-surface laser engraving [20], MLEM [21], wavelength discrimination [19,22], and combinations of each [23]. PSDs have the advantage of being able to measure DOI without any additional hardware, making them a cost-effective, practical, and expandable solution.

In a previous study, a Peak-to-Charge discrimination (PQD) method was developed to separate spontaneous alpha decay in LaBr_3_:Ce [24]. The PQD is a kind of pulse shape discrimination, which uses the Vp/Q (peak voltage, Vp, divided by the charge, Q) values of waveforms. Since decay constant of Gd_2_SiO_5_:Ce (GSO:Ce) depends on Ce dopant concentration [25], Vp/Q value also depends on the concentration. Therefore, PQD is expected to discriminate GSO:Ce scintillators with different Ce concentration.

The aim of the present study is to develop the PQD-based DOI data acquisition (DAQ) system and to evaluate its performance. The developed system provides online and low-latency DOI discrimination and can be integrated into PET.

## 2. Materials and Methods

### 2.1. Detector Module

**Figure 1:**
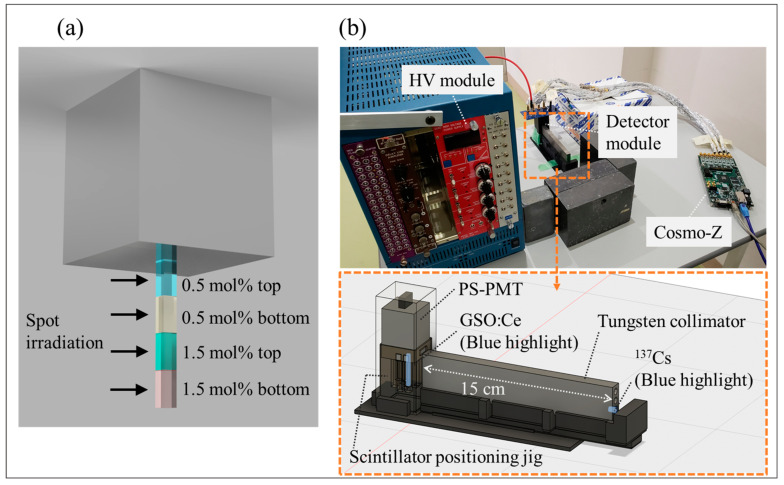
The experimental setup of the measurement. (**a**) shows the order of scintillators, and (**b**) shows the overview image. The scintillators were more distant from the PS-PMT with higher Ce-doped concentrations.

A detector was composed of four layers of GSO:Ce scintillators and a position-sensitive photomultiplier tube (PS-PMT; R-8900-00-C12, Hamamatsu Photonics K.K., Hamamatsu, Japan). The four crystals were obtained from both the top and bottom of ingots with a nominal Ce concentration of 0.5 mol% and 1.5 mol% (OXYDE Corporation, Hokuto, Japan). Decay time constants of the four crystals are shown in Table 1. The crystals were stacked in the order 0.5 mol% bottom, 0.5 mol% top, 1.5 mol% bottom, and 1.5 mol% top from the photocathode (Figure 1a). Each crystal was sized at 2.5 mm × 2.5 mm × 6 mm with chemically etched on side surfaces and polished on top and bottom side. The four-layer crystal block was wrapped with a reflector (22E6SR, TORAY Industries, Inc., Chuo-ku, Tokyo, Japan) except one face coupled to the PS-PMT with an optical grease (BC630, Saint-Gobain K.K., Chiyoda-ku, Tokyo, Japan). Since PMTs are considered more suitable for PQD due to their superior noise characteristics compared to SiPM [26,27], R8900-00-C12 was used in this study.

### 2.2. Electronics, Data Acquisition and Processing

Current-to-voltage conversion for the PS-PMT output signal is needed for the A/D conversion of the waveforms. Since the frequency response of the operational amplifier might have affected the waveform shape and thus the PQD performance, load resistance conversion was adopted instead of the trans-impedance amplifier (TIA). The larger the load resistance value, the larger the amplitude of the waveform, whereas the frequency response degrades, thus an appropriate load resistance value must be selected for the appropriate acquisition of waveforms. In this study, 10 kΩ load resistors were selected to ensure that the PS-PMT outputs do not exceed the voltage range of a later DAQ board and that the waveforms retain the frequency components required for the PQD. Similarly, if a weighted summing amplifier circuit was used to estimate the position of the luminescent scintillator on the PS-PMT photocathode, the PQD discrimination performance might be degraded due to the frequency response of the amplifier. Therefore, an anger-type logic circuit was employed for position estimation, and no operational amplifiers other than the ADC driver were used in this study. To keep the pulse height, a 10 Ω resistor chain was used. 

**Figure 2:**
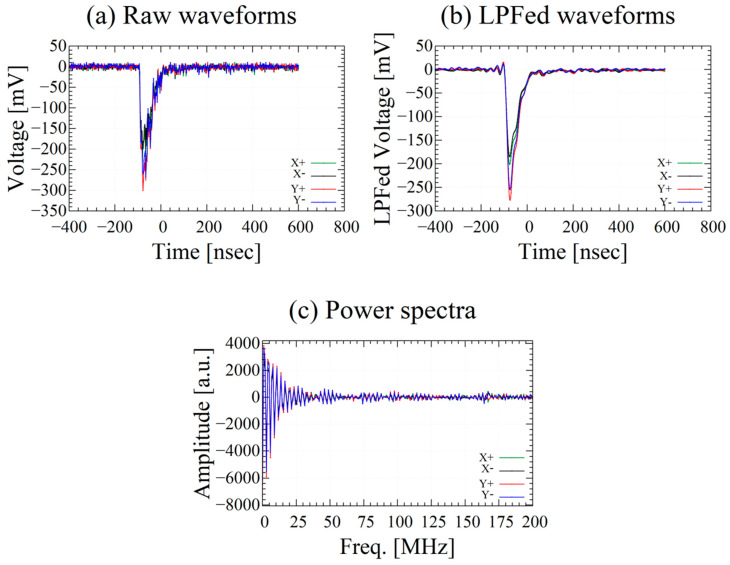
Output waveform of the phoswich detector measured with an oscilloscope. (**a**) waveform before applying LPF, (**b**) waveform after applying LPF, (**c**) frequency spectrum before applying LPF.

The PQD performance mainly depends on the accurate peak voltage acquisition of the waveform. Since developed DAQ system does not write waveforms to files, waveforms from aforementioned anger-type logic circuit acquired in a preliminary experiment using an oscilloscope (WaveRunner 64xi, Teledyne Japan Corporation, Fuchu, Japan) is shown in Figure 2 for reference. The output signals of the PS-PMT contain a lot of high-frequency noise, therefore a low-pass filter (LPF) is essential. The sampling rate and the bandwidth are also the dominant factors in determining the performance of the PQD since the accuracy of peak voltage acquisition affects the deviation of Vp/Q values. The PQD needs approximately 100 MSPS to discriminate Ce concentration difference of the GSO:Ce scintillators.

A Cosmo-Z board (TokushuDensiKairo Inc., Sumida-ku, Tokyo, Japan) has an 8ch 12-bit 100 MSPS Flash ADC connected with a Xilinx Zynq-7000 SoC. The bandwidth of the analog front-end (AFE) circuit is 50 MHz, the input voltage range is ±0.5 Vpp, and the input impedance is 50 Ω. Since arbitrary signal processing can be performed online by rewriting the Zynq-7000 firmware, this board was used as the DAQ board in this study. The Programmable Logic (PL) and the software working on the Processing System (PS) were developed in-house to specialize in the detector for the PQD. 

**Figure 3:**
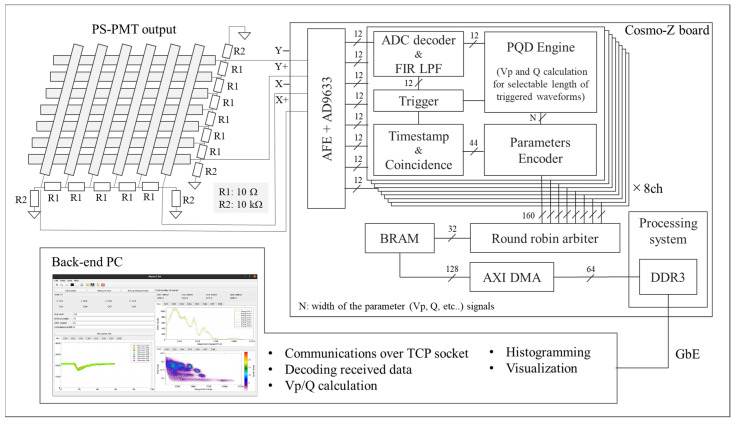
The overview of developed system. The outputs from PS-PMT were converted to digital signals by the Flash ADC on the Cosmo-Z and processed online at Programmable Logic. The processed data was transferred to a back-end PC for post-processing and visualization.

**Figure 4:**
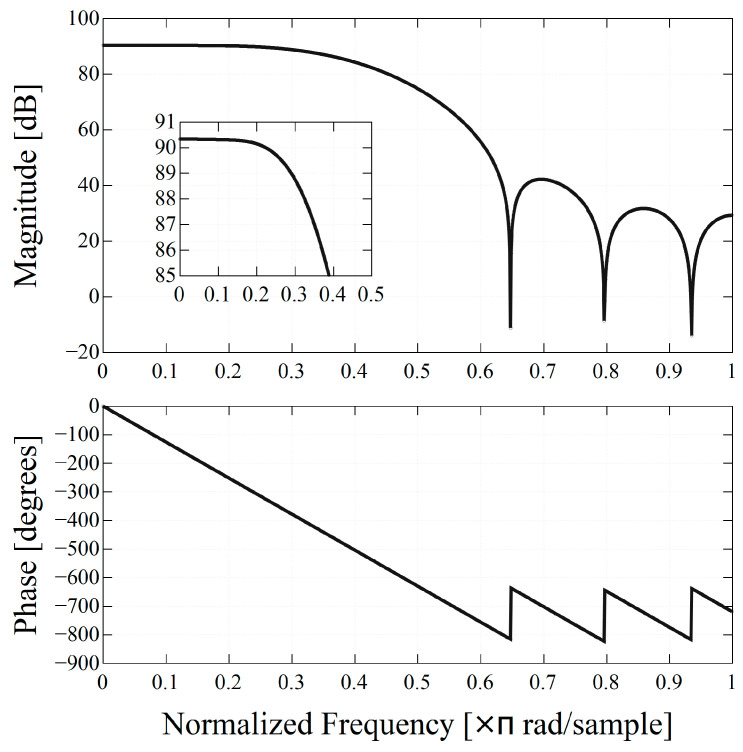
The frequency response of the 14th order FIR LPF implemented on the developed system. The cut-off frequency of the LPF was set at approximately 35 MHz.

The overview of the readout system and signal processing flow are shown in Figure 3. Output signals from the PS-PMT were divided into four signals, X+, X−, Y+, and Y− for position estimation. The four waveforms were amplified by a preamplifier and A/D was converted by the Flash ADC. Then the digitalized waveforms were sent to the 14thorder finite impulse response (FIR) LPF implemented on the Programmable Logic to acquire accurate peak voltage. The cut-off frequency of the LPF was set at approximately 35 MHz. The frequency response of the FIR filter is shown in Figure 4. The coefficients of the FIR filter to be implemented on FPGA must be integers, therefore, they were scaled by 2^15^ in this system. Since this made the outputs of the FIR filter 2^15^ times larger than the inputs, the outputs of the FIR filter were shifted 15 bits to the right, and 12 bits were extracted from the least significant bit (LSB) side. The waveforms applied to the LPF were sent to a trigger detector and a PQD engine. The trigger detector outputted a selectable length of the trigger signal for the waveforms exceeding the threshold level. The PQD engine calculated a Vp, Q, and pedestal during the trigger and a parameter encoder encoded them, combined with a 44-bit timestamp. The size of an event packet was 20 bytes. A round-robin arbiter read the event packets of each channel sequentially and stored them in Block RAM. An AXI DMA transferred the event packets from Block RAM to the SDRAM, and Processing System sent them to the back-end PC via Gigabit Ethernet (GbE). Graphical User Interface (GUI) software running on the back-end PC communicated with the Cosmo-Z board over a TCP socket, decoded the measurement data, calculated Vp/Q values, and performed histogram analysis and visualization.

### 2.3. Experimental Methods

The supply voltage for PS-PMT was set at −990 V and the output signals were acquired by the Cosmo-Z board with in-house firmware and software. The gain of the PS-PMT depends on the position on the photocathode, which is considered to affect the performance of PQD. To evaluate the PQD performance depending on the position of scintillators, 64 positions were assigned corresponding to an 8 × 8 array with a 2.7 mm pitch, and the scintillator block was shifted to each position for measurement. 

The performance of the DOI detector was compared between collimated and non-collimated irradiation. For the non-collimated irradiation, the 123.9 MBq ^137^Cs source was placed 15 cm away from the detector and 40,000 events were collected. For the collimated irradiation, the ^137^Cs source was placed at the far end of a 15 cm long tungsten collimator, 10,000 events were collected for each layer, and all layers were scanned. The experimental setup is shown in Figure 1b.

Jigs to position the scintillator block and the collimator were created by using a 3D printer (Anycubic Photon M3 Plus, Anycubic, Shenzhen, China).

### 2.4. Performance Evaluation

In accordance with the central limit theorem, 662 keV total absorption peaks of the energy histogram and Vp/Q values each follow a normal distribution. Since the Vp/Q value does not depend on deposit energy but on only the time constant of the scintillator [24], the 662 keV total absorption peak can be represented by a normal distribution of two variables, energy and Vp/Q value.

Energy resolutions for each layer were the FWHM obtained by fitting analysis with a one-dimensional normal distribution for the 662 keV total absorption peak of ^137^Cs.

**Figure 5:**
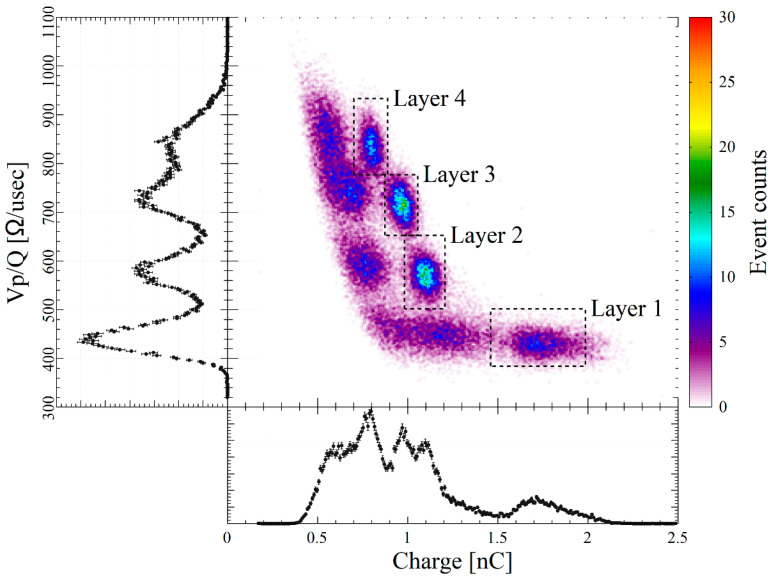
Q vs. Vp/Q 2D histogram with Q and Vp/Q 1D histogram. Dashed squares indicate 662 keV total absorption peaks of ^137^Cs. For 1D PQD, the events inside the squares were extracted and used for Vp/Q thresholding.

To determine the analysis range for the PQD and to evaluate the DOI discrimination performance, total absorption peaks were fitted with one-dimensional or two-dimensional normal distributions. Figure 5 shows the one-dimensional and two-dimensional histograms and their analysis ranges.

The boundaries of the analysis range for each crystal in the histograms are defined as

(1) $$B_{lxm}=\mu_{x}-2\sigma_{x},$$

(2) $$B_{hxm}=\mu_{x}+2\sigma_{x},$$

(3) $$B_{lym}=\left\{\begin{array}{c} I(m-1,m),\;\;\;if\;m>1\\ {\;\mu }_{y}-2\sigma_{y},\;\;if\;m=1 \end{array}\right.,$$

(4) $$B_{hym}=\left\{\begin{array}{c} I(m,m+1),\;\;\;if\;m<M\\ {\;\mu }_{y}+2\sigma_{y},\;\;if\;m=M \end{array}\right.,$$

where $B_{lxm}$ and $B_{hxm}$ are the lower and higher boundaries for *m*-th crystal along *x*-axis, respectively, $B_{lym}$ and $B_{hym}$ are the lower and higher boundaries for m-th crystal along *y*-axis, respectively, $\mu_{x}$ and $\mu_{y}$ are the mean values of *x* and *y* of the distribution, respectively, $\sigma_{x}$ and $\sigma_{y}$ are the standard deviations of *x* and *y* of the distribution, respectively, and I(a,b) is the intersection of the a-th and b-th normal distributions, which is the point between the two averages. The Vp/Q histograms for DOI discrimination performance were calculated by extracting events inside the boundaries (1)–(4).

**Figure 6:**
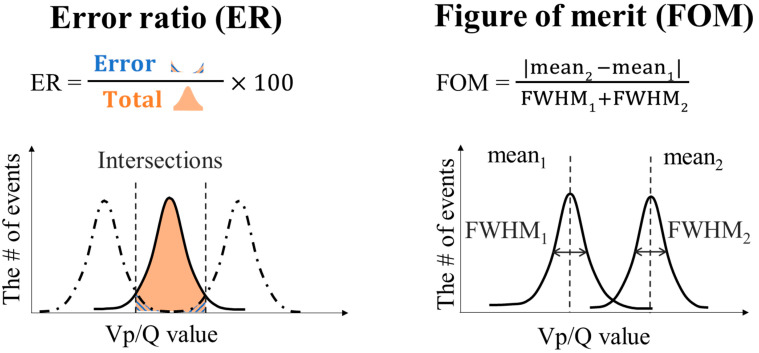
Illustration of the one-dimensional error ratio (ER) and figure of merit (FOM).

*ER_nD_* (Error Ratio) and *FOM_nD_* (Figure of Merit) were defined as *n*-dimensional DOI identifiability by fitting with *n*-dimensional normal distribution. *ER_nD_*, which is the discrimination ability of each crystal, is computed as

(5) $$ER_{nD}=\frac{n_{EnD}}{n_{TnD}},$$

where $n_{EnD}$ is the number of error events occurred at the other crystals and $n_{TnD}$ is the number of total events inside the boundaries. *FOM_nD_*, which is the discrimination ability between 2 crystals, is computed as

(6) $${FOM}_{nD}=\frac{\left\|{mean}_{nDb}-{mean}_{nDa}\right\|}{{FWHM}_{nDb}+{FWHM}_{nDa}},$$

where *mean_nDa_* is the mean value of one, *mean_nDb_* is the mean value of the other, *FWHM_nDa_* is the *FWHM* of one, and *FWHM_nDb_* is the *FWHM* of the other. Figure 6 shows an illustration of the one-dimensional ER and FOM.

Calculations of the $n_{EnD}$, $n_{TnD}$ and ${FWHM}_{2Da,b}$ are given in the Appendix A.

## 3. Results

**Figure 7:**
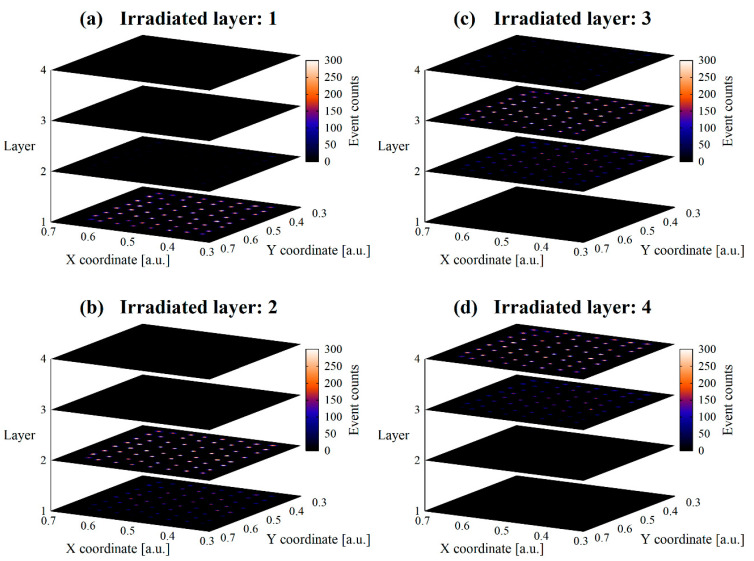
Flood histograms obtained by collimated irradiation. In (**a**), no events are observed in the non-irradiated layer, while in (**b**–**d**), few leakage events existed in the PMT-side layer. This indicates that the leakage events are due to the experimental setup, not crosstalk.

A flood histogram acquired by the collimated irradiation is shown in Figure 7. It shows that the targeted layer is irradiated accurately. Some leakage events are observed in the layer on the photocathode side, however, few leakage events existed in the layer on the opposite side. Therefore, these leakages are due to the collimator diameter and experimental setup, not due to crosstalk.

**Figure 8:**
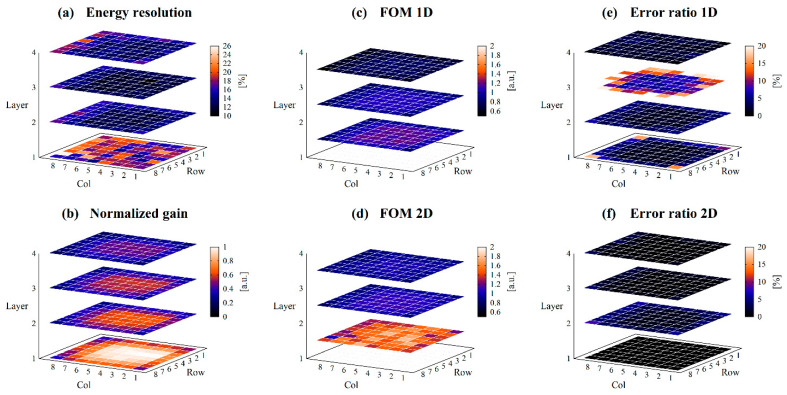
Detector performances obtained with collimated irradiation. (**a**) shows the energy resolution, (**b**) shows the normalized gain, (**c**) shows the FOM_1D_, (**d**) shows the FOM_2D_, (**e**) shows the ER_1D_, and (**f**) shows the ER_2D_ for each position of scintillator block.

**Figure 9:**
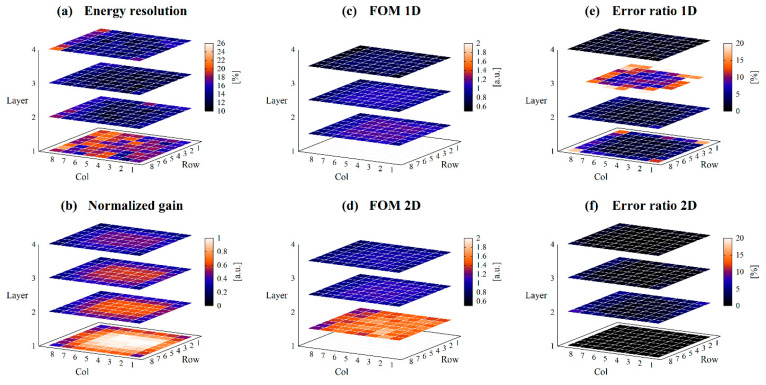
Detector performances obtained with non-collimated irradiation. (**a**) shows the energy resolution, (**b**) shows the normalized gain, (**c**) shows the FOM_1D_, (**d**) shows the FOM_2D_, (**e**) shows the ER_1D_, and (**f**) shows the ER_2D_ for each position of scintillator block.

**Figure 10:**
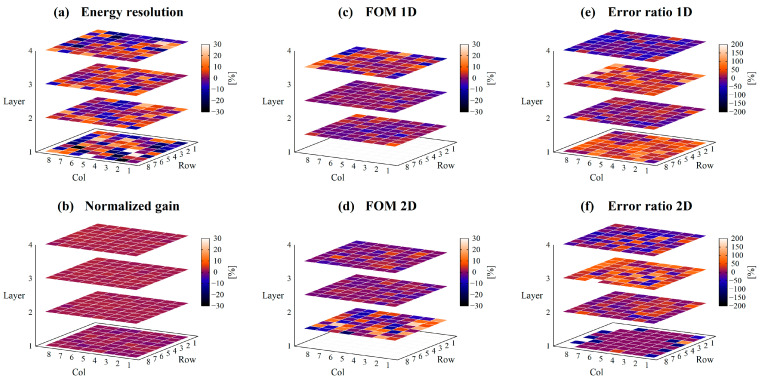
Difference of the detector performances between collimated and non-collimated irradiation. (**a**) shows the energy resolution, (**b**) shows the normalized gain, (**c**) shows the FOM_1D_, (**d**) shows the FOM_2D_, (**e**) shows the ER_1D_, and (**f**) shows the ER_2D_ for each position of scintillator block.

While Figure 8 shows the results for collimated irradiation, Figure 9 shows the results for non-collimated irradiation, and Figure 10 shows the differences. The mean values and SDs of the performance index for each layer are summarized in Table 2.

The energy resolution of the first layer was worse than the other layers, approximately 18% on average. The other layers had an average energy resolution of 12.4–14.4%. The difference in mean values of energy resolution with and without collimation was approximately 2%.

The normalized gain was higher in the center of the photocathode or closer to the photocathode and lower at the edge of the photocathode or farther from the photocathode. There was little difference in normalized gain with or without collimation.

FOMs were higher and ERs were lower for crystals closer to the photocathode, which means that crystals closer to the photocathode have better DOI discrimination ability. Since each crystal layer has a different 662 keV peak position due to the difference in the amount of light reaching the PS-PMT, two-dimensional discrimination using both Q and Vp/Q values showed improved discrimination performances.

The differences in FOMs with and without collimation were slight, within 1.2%, however, the differences in ERs appeared to be large, up to approximately 54%. This is due to the very small values of ERs.

## 4. Discussion

The DOI detector system based on the PS-PMT was developed by using the PQD. Less than 2.2% differences with and without collimation were shown for the mean values of detector performances other than ERs. Therefore, collimation does not affect the PQD discriminability.

The first layer showed worse energy resolution than the other layers. Optical photons generated in each layer travel in two paths, one heading directly to the PMT, and the other traveling to the fourth layer and then being reflected back to the PMT. Photons generated in the first layer of the scintillator tend to have worse energy resolution due to the large difference in the length of these two paths. However, the energy resolution of the detector system developed in this study is comparable to previous studies [9,10,12,13,14,15,17,18,20,22,23] and is considered sufficient for DOI-PET for small animals.

The results showed that the mean FOMs_1D_ are 1.5, 0.99, 0.91 for layers between 1st-2nd, 2nd-3rd, and 3rd-4th respectively, and the mean ERs_1D_ are 3.50%, 2.96%, 13.3%, and 1.88% for layers 1st, 2nd, 3rd, and 4th, respectively. Since they are comparable to the previous studies [9,10,20,23], their performance is considered to meet the required performance for a DOI detector.

In this study, a 2D PQD was introduced that utilizes the unique property of the phoswich detector to have different normalized gains for each layer. Although the ERs_1D_ of the third layer was relatively poor, the 2D PQD resulted in the mean ERs_2D_ of less than 3% and the mean FOMs_2D_ of more than 0.9 for all layers. Further optimization of the decay constant and crystal position might improve the discrimination ability by increasing the 2D Mahalanobis distance in Q vs. Vp/Q 2D histogram. The optimization might include the material of the optical grease.

The higher the normalization gain, the higher the DOI discrimination performance was observed. This might be caused by the improved accuracy in obtaining Vp and smaller variation in Vp/Q as the gain increases. Using the larger value load resistor for current-to-voltage conversion was expected to increase the gain and to improve PQD performance, however, the input range of the Cosmo-Z and the scintillator position dependency of the gain of PS-PMT make changing the resistor value difficult. The input range of the Cosmo-Z is ±500 mV, and the pedestal is approximately 0 mV. The pulse height at the high gain position of the PS-PMT is about 400 mV, which is near the limit of the input range of the Cosmo-Z. If a larger load resistor is used, the waveform might be saturated. On the other hand, at the low gain position of the PS-PMT, such as the edge of the photocathode, the 662 keV total absorption peak is likely to be buried in noise due to low pulse height. Thus, using lower load resistor seems to be impractical. Therefore, designing a low-noise, wide-bandwidth trans-impedance amplifier that amplifies the PMT output to barely the input range of the Cosmo-Z might improve PQD performance. Optimization of the voltage divider circuit for position estimation might also contribute to improved performance. However, in this study, the scintillators were not arranged in an array. The scintillator array might have caused performance degradation due to crosstalk.

Since the PQD is a kind of PSD, the crystal shape is not constrained. Some previous studies [28,29] propose unique arrangements of the crystals such as taper shape to improve the detection efficiency. The detector system developed in this study could be implemented for such arrangements. Additionally, a previous study [23] combined PSD and LS to achieve four layer discrimination. Since PQD is a kind of PSD and is considered to be unaffected by reflective materials, PQD could also be combined with LS or laser engraving techniques. It might allow for more multi-layered discrimination.

The count rate when the source was placed close to the detector without the collimator was saturated at approximately 12 kcps. Since a single event was measured on four channels, the maximum event rate that can be processed by the developed system was approximately 3 k events/sec. The mass attenuation coefficient of GSO is 0.0814 cm^2^/g for 662 keV and the density of GSO is 6.7 g/cm^3^, thus the probability of interaction with 2.5 mm thickness GSO:Ce is 12.7%. The geometrical efficiency with the tungsten collimator was 6.95 × 10^−3^%. Therefore, the detection efficiency of collimated measurement was 8.82 × 10^−4^% and the event rate with 123.9 MBq ^137^Cs source is 1 k events/sec. It means that counting losses might occur in PET applications with a higher count rate although all events could be measured for the collimated experiment in this study. The signal processing circuit implemented on the Programmable Logic was pipelined and running at 100 MHz, which is the same as the sampling frequency of the Flash ADC, and the event processing rate of the Programmable Logic was 5 M events/sec. Since the DMA bandwidth is also larger than the Gigabit Ethernet (GbE) data transfer bandwidth [30], GbE communication and postprocessing for visualization are expected to be the rate-limiting factor. The back-end PC was equipped with 8GB of RAM and Intel Core i5-5278U CPU (2.9 GHz × 4 cores). The mean software processing time and SD were measured to be 99.07 ± 16.17 ms for socket communication, 37.94 ± 2.477 ms for decoding, 3.352 ± 10.09 ms for histogram calculation, and 97.90 ± 7.029 ms for visualization. A DAQ developed in a previous study has processed 250 k events/sec [31], and compared to this, the system developed in this study is slow. However, the processible data rate improvement is out of the scope of this study. The histogram calculation and visualization can be omitted for some applications, although socket communication and decoding are required. Thus, to achieve higher processible event rates, omitting post-processing for visualization, hard-coded communication, full FPGA implementation of PQD, event packet reduction, and improvement of back-end PC performance would be effective. These improvements should be taken for future work.

In this study, 662 keV gamma rays from ^137^Cs source were measured. In detecting 511 keV gamma rays for PET applications, a large load resistor for current-to-voltage conversion might be needed since the lower pulse height than that of 662 keV gamma rays might reduce the PQD performance. LSO:Ce, which is one of the most commonly used scintillator for PET, has approximately four times higher light yield than GSO:Ce. Therefore, if LSO:Ce is used with the developed system in this study to achieve more layers of scintillators, gain adjustment might be required. The developed system could discriminate it since the decay time of LSO:Ce is approximately 40 ns.

In this study, the PQD-based DOI-PET detector system was developed. The admirable AFE circuit of the Cosmo-Z and the FIR filter implemented in the Programmable Logic showed good discrimination ability as DOI detector even using the 1D PQD. Also, the 2D PQD introduced in this study showed more accurate DOI discriminations. Therefore, more multi-layered detectors could be implementable. Since the array detectors were not used in this study, the effect of crosstalk could not be evaluated. Future work will evaluate the PQD performance with a multi-layered GSO:Ce array. This system could be applied to various PSD applications since the PQD is simple and has few restrictions on detectors.

## Figures and Tables

**Table 1:** Decay time constants of GSO:Ce scintillators used for the phoswich detector.

Ce Concentration [mol%]	Primary Decay Time Constant [ns]	Secondary Decay Time Constant [ns]
0.5 (top)	61.3	1135
0.5 (bottom)	41.3	608
1.5 (top)	27.3	266
1.5 (bottom)	23.4	239

**Table 2:** The mean values and SDs of each detector performance indices with and without collimation.

	Layer	Collimated		Non-Collimated		Difference of Means [%]
		Mean	SD	Mean	SD	
Energy resolution [%]	1st	18.22	2.36	17.84	1.85	2.11
	2nd	13.62	1.46	13.82	1.60	−1.41
	3rd	12.43	1.12	12.69	1.13	−2.01
	4th	14.41	1.81	14.27	1.76	0.94
Normalized gain [a.u.]	1st	0.73	0.18	0.74	0.18	−0.71
	2nd	0.47	0.11	0.48	0.12	−1.70
	3rd	0.42	0.10	0.43	0.11	−1.96
	4th	0.34	0.08	0.35	0.09	−2.04
FOM_1D_ [a.u.]	1st-2nd	1.04	0.11	1.03	0.10	0.50
	2nd-3rd	0.95	0.09	0.95	0.09	0.35
	3rd-4th	0.70	0.07	0.71	0.07	−1.09
FOM_2D_ [a.u.]	1st-2nd	1.46	0.15	1.48	0.13	−1.16
	2nd-3rd	0.99	0.08	0.99	0.09	0.49
	3rd-4th	0.91	0.06	0.91	0.07	0.44
ER_1D_ [%]	1st	3.50	3.32	4.40	3.43	−20.54
	2nd	2.96	1.27	2.79	1.20	5.97
	3rd	13.30	9.50	17.31	14.00	−23.14
	4th	1.88	0.67	1.30	0.42	44.37
ER_2D_ [%]	1st	0.00	0.00	0.00	0.00	−54.07
	2nd	2.73	1.48	2.80	1.48	−2.37
	3rd	0.88	0.68	1.22	1.02	−27.98
	4th	0.65	0.68	0.52	0.49	24.27

## Data Availability

Data is contained within the article.

## Appendix A

When the detector consists of the M layered Gd_2_SiO_5_ (GSO) crystals, M one-dimensional normal distributions, $f_{m}(x)$, appear in the one-dimensional Vp/Q histogram. Additionally, M two-dimensional normal distributions, $f_{m}(x,y)$, appear in the two-dimensional histogram for Q and Vp/Q.

The boundary of each crystal in the histograms is defined as

(A1) $$B_{lxm}=\mu_{x}-2\sigma_{x},$$

(A2) $$B_{hxm}=\mu_{x}+2\sigma_{x},$$

(A3) $$B_{lym}=\left\{\begin{array}{c} I(m-1,m),\;\;\;if\;m>1\\ {\;\;\mu }_{y}-2\sigma_{y},\;if\;m=1 \end{array}\right.,$$

(A4) $$B_{hym}=\left\{\begin{array}{c} I(m,m+1),\;\;\;if\;m<M\\ {\;\;\mu }_{y}+2\sigma_{y},\;if\;m=M \end{array}\right.,$$

where $B_{lxm}$ and $B_{hxm}$ are the lower and higher boundaries for *m*-th crystal along *x*-axis, respectively, $B_{lym}$ and $B_{hym}$ are the lower and higher boundaries for *m*-th crystal along *y*-axis, respectively, $\mu_{x}$ and $\mu_{y}$ are the mean values of *x* and *y* of the distribution, respectively, $\sigma_{x}$ and $\sigma_{y}$ are the standard deviations of *x* and *y* of the distribution, respectively, and I(a,b) is the intersection of the *a*-th and *b*-th normal distributions, which is the point between the two averages.

For one-dimensional normal distribution, the number of error events occurred at the other crystals $n_{E1D}$ and the number of total events inside the boundaries $n_{T1D}$ are computed as

(A5) $$n_{E1Dm}=n_{T1Dm}-\int_{B_{lxm}}^{B_{hxm}}f_{m}(x)dx,$$

(A6) $$n_{T1Dm}=\sum_{i=1}^{M}\int_{B_{lxm}}^{B_{hxm}}f_{i}(x)dx.$$

For two-dimensional normal distribution, $n_{E2D}$ and $n_{T2D}$ are computed as

(A7) $$n_{E2Dm}=n_{T2Dm}-\int_{B_{lxm}}^{B_{hxm}}\int_{B_{lym}}^{B_{hym}}f_{m}\left(x,y\right)dx\;dy,$$

(A8) $$n_{T2Dm}=\sum_{i=1}^{M}\int_{B_{lxm}}^{B_{hxm}}\int_{B_{lym}}^{B_{hym}}f_{i}\left(x,y\right)dx\;dy.$$

The probability density function of the two-dimensional normal distribution, $P_{r}\left(x,y\right)$, is

(A9) $$P_{r}\left(x,y\right)=\frac{1}{2\pi \sigma_{x}\sigma_{y}\sqrt{1-\rho^{2}}}\exp\left[-\frac{1}{2\left(1-\rho^{2}\right)}\left\{{\left(\frac{x-\mu_{x}}{\sigma_{x}}\right)}^{2}-2\rho \left(\frac{x-\mu_{x}}{\sigma_{x}}\right)\left(\frac{y-\mu_{y}}{\sigma_{y}}\right)+{\left(\frac{y-\mu_{y}}{\sigma_{y}}\right)}^{2}\right\}\right],$$

where ρ is the correlation between x and y.

For *n*-dimensional normal distribution, the probability density function is determined by the Mahalanobis distance d. For two-dimensional normal distribution, Mahalanobis distance is
  (A10) $$d=\sqrt{\frac{1}{\left(1-\rho^{2}\right)}\left[{\left(\frac{x-\mu_{x}}{\sigma_{x}}\right)}^{2}-2\rho \left(\frac{x-\mu_{x}}{\sigma_{x}}\right)\left(\frac{y-\mu_{y}}{\sigma_{y}}\right)+{\left(\frac{y-\mu_{y}}{\sigma_{y}}\right)}^{2}\right]}.$$
  Since the square of Mahalanobis distance follows the chi-squared distribution with *n* degrees of freedom, the cumulative distribution function of two-dimensional normal distribution, $F\left(d\right)$, is
  (A11) $$F\left(d\right)=1-\exp\left[-\frac{d^{2}}{2}\right].$$
  Thus, when HWHM_2D_ is expressed in Mahalanobis distance, $d_{HWHM_{2D}}$, (A11) becomes
  (A12) $$\frac{1}{2}=1-\exp\left[-\frac{d_{HWHM_{2D}}^{2}}{2}\right],$$
  (A13) $$d_{HWHM_{2D}}=\sqrt{2ln(2)}.$$
  From (A11) and (A13), the HWHM_2D_ for arbitrary angle φ is calculated as
  (A14) $$r=HWHM_{2D}\left(\varphi \right)=\sqrt{\frac{2\left(1-\rho^{2}\right)\cdot \ln\left(2\right)}{1-2\rho \sin\varphi \cos\varphi }},$$
  where
  (A15) $$r=\sqrt{D_{x}^{2}+D_{y}^{2}},$$
  (A16) $$D_{x}=\frac{x-\mu_{x}}{\sigma_{x}},$$
  (A17) $$D_{y}=\frac{y-\mu_{y}}{\sigma_{y}}.$$
  Thus, ${FWHM}_{2D}$ for arbitrary angle φ is
  (A18) $${FWHM}_{2D}\left(\varphi \right)=2\times HWHM_{2D}\left(\varphi \right).$$
